# Carcinome muco-épidermoide chez une jeune fille de 21 ans

**DOI:** 10.11604/pamj.2015.20.6.5073

**Published:** 2015-01-05

**Authors:** Fatima Zahra Khouchilia, Wiam El khattabi, Régis Gothard Bopaka, Abdelaziz Aichane, Hicham Afif

**Affiliations:** 1 Service des Maladies Respiratoires, Hôpital 20 Août, CHU Ibn Rochd, Casablanca, Maroc

**Keywords:** Carcinome, mucoépidermoide, bronchoscopie, pneumonectomie, carcinoma, muco-epidermoid, bronchoscopy, pneumonectomy

## Abstract

Les carcinomes muco-épidermoïdes font partie d'un groupe rare de tumeurs pulmonaires malignes. Ces tumeurs sont le plus souvent retrouvées chez lessujets jeunes. La croissance de la tumeur est en général endo-bronchique et concerne les bronches de grande taille. L'aspect histo-pathologique révèle des cellules produisant du mucus, des cellules épithéliales et des cellules mixtes. Dans cet article nous rapportons l'observation d'une jeune fille de 21 ans avec une tumeur de la bronche souche droite, qui après résection endo-bronchique s'est avéré être un carcinome muco-épidermoïde pulmonaire. La patiente a subi une pneumonectomie droite élargie à la carène avec un curage ganglionnaire médiastinale. Elle est actuellement en rémission après un suivi de 7 mois. Aucun traitement standard n'est défini pour ces tumeurs. Le pronostic dépend du grade histologique, et peut, notamment chez les sujets âgés, être très péjoratif.

## Introduction

Le carcinome muco-épidermoïde bronchique est une tumeur maligne survenant habituellement au niveau des glandes salivaires [1]. Elle est très rare au niveau du thorax et constitue de 0,1 à 0,2% des tumeurs pulmonaires malignes [1–3]. L’âge de survenue est compris entre trois mois et 78 ans avec une moyenne d’âge de 40 ans. Le traitement de choix en est l'ablation chirurgicale. La résection complète des tumeurs de bas grade de malignité assure un pronostic favorable; par contre les tumeurs de haut grade de malignité donnent plus souvent une récidive locale associée à un risque métastatique élevé.

## Patient et observation

Il s'agit de Mlle XY, âgée de 21 ans, n'a pas d'habitudes toxiques et n'a pas d'antécédent néoplasique personnel ou familial. Elle s'est présentée à la consultation pour une douleur thoracique droite remontant à 3 mois et résistante aux traitements antalgiques usuels, associée à une dyspnée à l'effort, une toux sèche et 2 épisodes d'hémoptysie. Le tout évoluait dans un contexte d'amaigrissement chiffré à 9kg en 3mois.

**Figure 1:**
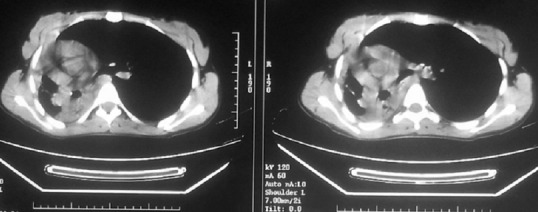
Tomodensitométrie thoracique objectivant un trouble de ventilation droit

**Figure 2:**
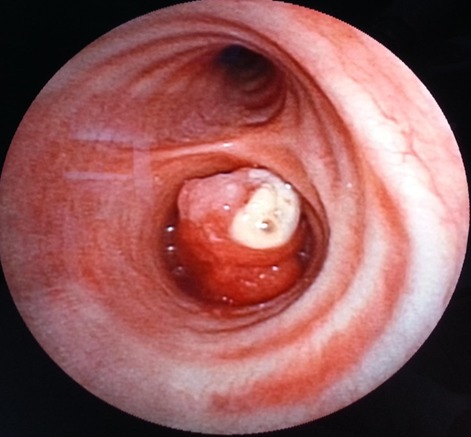
Tumeur à couleur rougeâtre, recouverte de nécrose et obstruant totalement l'entrée de la bronche souche droite

La tomodensitométrie (TDM) thoracique a révélé un trouble de ventilation droit avec une attraction des éléments de médiastin vers le même coté (Figure 1). Labronchoscopie souple a objectivé la présence d'un bourgeon d'allure tumorale, à surface très vascularisée faisant glisser la pince de la biopsie, recouvert de nécrose et obstruant totalement l'entrée de la bronche souche droite (Figure 2). La biopsie de ce bourgeon était très délicate vu le risque hémorragique. Par conséquent, l'analyse histo-pathologique de ces prélèvements était non contributive. Afin de confirmer la nature histologique de ce bourgeon dont l'origine tumorale est très probable, nous avons opté pour une thoracotomie à visée diagnostique et thérapeutique. Le bilan d'extension réalisé en préopératoire était normal. La thoracotomie est pratiquée sous anesthésie générale, par voie postéro-basale, passant par le 5^ème^ espace intercostal droit, et a trouvé un poumon complètement hépatisé siège de dilatation kystique et des formations nodulaires diffuses avec une tumeur proximale bronchique de la bronche souche droite.

L'intervention a consisté en une pneumonectomie droite élargie à la carène avec une réimplantation et une anastomose termino-terminale bronche principale gauche et trachée avec un curage ganglionnaire médiastinale, intertrachéobronchique, loge de barety et artère pulmonaire. Les résultats des analyses histopathologiques de la pièce d'exérèse ont montré un Carcinome muco-épidermoïde pulmonaire (de type tumeur glandes salivaires), intrabronchique proximal de 3,8 cm de grand axe, sans envahissement du hile ni de la plèvre viscérale et sans métastases ganglionnaires médiastinales.

Le diagnostic retenu en post opératoire était en faveur d'un carcinome bronchique non àpetite cellule de type histologique muco-épidermoïde classé de bas grade de malignité La patiente est actuellement en rémission après un suivi de 7 mois.

## Discussion

Le carcinome muco-épidermoïde bronchique a été décrit pour la première fois en 1952 par Smetana et al [3–7], mais il faut prendre en compte que les moyens diagnostiques des années 50 ne permettaient sans doute pas la distinction entre tumeur muco-épidermoide primitive ou secondaire. Depuis, on retrouve des descriptions de cas isolés ou rassemblés en petites séries. Environ 1 à 5% des adénomes endobronchiques se révèlent être des carcinomes muco-épidermoïdes [3–8]. Dans la majorité des cas, comme décrit ci-dessus, on retrouve ce type de néoplasie dans les voies respiratoires de grande taille: les bronches souches, bronches lobaires, bronches segmentaires; on les retrouve exceptionnellement dans l'arbre bronchique distal [3–9]. Dans notre étude le carcinome muco-épidermoïde est retrouvé dans la bronche souche droite.

Les symptômes observés sont liés à l'obstruction des voies aériennes et peuvent se présenter sous forme de pneumonie, toux chronique, dyspnée, sifflement ou hémoptysie [1, 3–8]. Dans la littérature, on signale parfois la coexistence de cette maladie avec une agénésie congénitale d'un lobe pulmonaire [3, 10]. Notre patiente a présenté une toux sèche, une dyspnée à l'effort et deux épisodes d'hémoptysie.

A cause de la localisation de la tumeur, le plus souvent centrale, la radiographie standard ne montre habituellement aucun signe d'anomalie jusqu’à l'apparition de complications comme une pneumonie lobaire ou une atélectasie [1].

A l'examen tomodensitométrique, on peut découvrir une opacité ovale, bien limitée, d'aspect lobulaire; le rehaussement des nodules après injection de produit de contraste est plutôt modéré et on peut y retrouver des calcifications punctiformes [3–10]. Le carcinome muco-épidermoïde est rare au niveau bronchique, de ce fait, il faut prendre en compte la possibilité de la présence de métastases pulmonaires des tumeurs primitives des glandes salivaires. Dans notre cas, la TDM thoracique a montré un trouble de ventilation droit avec une attraction des éléments de médiastin vers le même coté.

Compte tenu de la localisation, l'examen diagnostique de choix est la bronchoscopie souple [3–6]. Néanmoins, puisque la localisation est très souvent sous-muqueuse et que l'aspect histologique ressemble aux autres pathologies endobronchiques, le diagnostic basé sur des prélèvements superficiels peut donner des renseignements imprécis. La rareté des tumeurs, l'anamnèse, l'aspect macroscopique et la taille limitée des biopsies peuvent influencer le diagnostic posé par l'anatomo-pathologiste.

On différencie deux formes histologiques du carcinome muco-épidermoïde du poumon. Dans 95% de cas, ce sont des tumeurs de bas grade de malignité. Elles se caractérisent par un taux plutôt faible de récidives locales et systémiques; le taux de survie à cinq ans dans ce groupe est de 80%. La forme de haut grade de malignité dont le taux de survie à cinq ans est de 30%, est souvent difficile à différencier des cancers bronchiques adénosquameux [3–10].

L'ablation chirurgicale de la tumeur semble être le moyen de traitement le plus efficace pour ce type de néoplasie et permet d'atteindre un haut niveau de survie (de plusieurs années) sans récidive locale et sans métastases à distance [3–8].

Les tumeurs de bas grade doivent faire l′objet dans la mesure du possible d′un traitement conservateur, à l′inverse des tumeurs de haut grade d′évolution péjorative [2]. Dans notre cas, vu la localisation intrabronchique très proximale de la tumeur, la patiente a bénéficié d'une pneumonectomie droite élargie à la carène, complétée par un curage ganglionnaire médiastinal, même si elle est classée de bas grade de malignité.

Jusqu’à présent, il n'y a pas de données scientifiques suffisantes permettant de confirmer l'efficacité du traitement du carcinome muco-épidermoïde du poumon par radiothérapie et chimiothérapie.

## Conclusion

Les carcinomes muco-épidermoides sont des tumeurs endo-bronchiques rares. La présentation radio-clinique est non spécifique. Leur diagnostic difficile sur des biopsies de taille limitée, est souvent porté après thoracotomie pour suspicion d'autres types de carcinome bronchique non à petites cellules. Le pronostic est variable selon le grade histologique.
